# Biocontrol of *Aspergillus flavus* on Peanut Kernels Using *Streptomyces yanglinensis* 3-10

**DOI:** 10.3389/fmicb.2018.01049

**Published:** 2018-05-23

**Authors:** Qaiser Shakeel, Ang Lyu, Jing Zhang, Mingde Wu, Guoqing Li, Tom Hsiang, Long Yang

**Affiliations:** ^1^State Key Laboratory of Agricultural Microbiology and Hubei Key Laboratory of Plant Pathology, Huazhong Agricultural University, Wuhan, China; ^2^School of Environmental Sciences, University of Guelph, Guelph, ON, Canada

**Keywords:** *Streptomyces yanglinensis* 3-10, peanut kernels, *Aspergillus flavus*, aflatoxin, biological control

## Abstract

The bacterium, *Streptomyces yanglinensis* 3-10, shows promise in the control of many phytopathogenic fungi. In this study, *S. yanglinensis* and its antifungal substances, culture filtrate (CF^3-10^) and crude extracts (CE^3-10^), were evaluated for their activity in reducing growth and aflatoxin AFB_1_ production by *Aspergillus flavus*, both *in vitro* and *in vivo* on peanut kernels. The results showed that in dual culture conditions, *S. yanglinensis* reduced the mycelial growth of *A. flavus* about 41% as compared to control. The mycelial growth of *A. flavus* was completely inhibited on potato dextrose agar amended with CF^3-10^ at 3% (v/v) or CE^3-10^ at 2.5 μg/ml. In liquid culture experiments, growth inhibition ranged from 32.3 to 91.9% with reduction in AFB_1_ production ranging from 46.4 to 93.4% using different concentrations of CF^3-10^ or CE^3-10^. For *in vivo* assays, CF^3-10^ at 0.133 ml/g (v/w) or CE^3-10^ at 13.3 μg/g (w/w) reduced the postharvest decay of peanut kernels by inhibiting visible growth of *A. flavus* leading to an 89.4 or 88.1% reduction in AFB_1_ detected, respectively. Compared with the controls, CF^3-10^ and CE^3-10^ in *A. flavus* shake culture significantly reduced expression levels of two AFB_1_ biosynthesis genes, *aflR* and *aflS*. Furthermore, electron microscopy observation showed that CF^3-10^ (2%, v/v) caused hyphae growth to be abnormal and shriveled, cell organelles to degenerate and collapse, large vacuoles to appear. These results suggest that *S. yanglinensis* 3-10 has potential as an alternative to chemical fungicides in protecting peanut kernels and other agricultural commodities against postharvest decay from *A. flavus*.

## Introduction

*Aspergillus flavus* is an important pathogenic fungus affecting peanuts during storage (Amaike and Keller, 2011). In infected peanuts it can cause seed rot and reduce seed viability and germination (Kumar et al., 2008). The pathogen can also produce aflatoxins, a group of fungal secondary metabolites, which are the most toxic carcinogens among known mycotoxins (Calvo et al., 2002; Klich, 2007; Krishnamurthy et al., 2008). These metabolites are considered potent hepatocarcinogens in animals and may also be involved in primary liver cancer and other cancers in kidney, lung, and colon tissues in humans (Bullerman, 1976; Amaike and Keller, 2011). The gene cluster for aflatoxin biosynthesis is 70 kb in length, containing 25 genes (Yu et al., 2004). In the aflatoxin biosynthesis gene cluster, *aflR* and *aflS* are the most important regulatory genes affecting the expression of aflatoxin biosynthesis genes (Yu et al., 2004; Amaike and Keller, 2011). The *aflR* gene encodes a zinc finger transcription factor, which binds to the consensus sequence 5′-TCGN_5_CGA-3′ in the promoter region of the aflatoxin biosynthetic genes, and positively regulates the transcription of these genes (Yu et al., 2004; Amaike and Keller, 2011). Another regulatory gene *aflS* (originally named *aflJ*) regulates aflatoxin production through direct interaction with *aflR* (Chang, 2004; Du et al., 2007).

To protect food and feedstuffs and to avoid aflatoxin contamination from *A. flavus*, there are several possible control strategies employed in peanut such as treating peanuts with fungicides, fumigants, or biocontrol agents (Kong et al., 2010). Although the use of fungicides for controlling *A. flavus* is an effective measure, there are several negative effects of this method including generation of fungicide-resistant strains of *A. flavus*, and fungicide residues affecting food safety and as a source of environmental pollution (Droby, 2006). Therefore, development of effective biological controls is a priority with presumed lower risk to humans and the environment.

Microorganisms have gained considerable attention in recent years because of their diversity and biological activities, mainly due to their ability to produce novel chemical compounds of high commercial value (Amador et al., 2003). As prokaryotes, *Streptomyces* spp. are Gram-positive bacteria that grow extensively in soil, on plants, and in air dust (Demain, 1999; Kettleson et al., 2013; Supong et al., 2016), and are known to produce over 7,500 bioactive compounds including anticancer agents, vitamins, and antibiotic compounds (Berdy, 2005; Gallagher et al., 2010; Manivasagan et al., 2014). Many *Streptomyces* species have been successfully evaluated as biocontrol agents against phytopathogens (Berg et al., 2001; Minuto et al., 2006; Law et al., 2017). Some antibiotic compounds (Al-Bari et al., 2007; Zucchi et al., 2008; Lyu et al., 2017) and other compounds such as chitinases (Gomes et al., 2001; Mander et al., 2016) produced by *Streptomyces* spp. have a strong antagonistic effect on growth and development of *Aspergillus*. Furthermore, some antibiotics, such as Blasticidin A (Sakuda et al., 2000a,b) and Dioctatin A (Yoshinari et al., 2007), produced by *Streptomyces*, can inhibit aflatoxin production in *Aspergillus*. Our previous study showed that crude extracts from liquid cultures of *Streptomyces yanglinensis* isolate 3-10 had high antifungal activity against several plant pathogenic fungi including *A. flavus* (Lyu et al., 2017). Therefore, the biocontrol potential of *S. yanglinensis* isolate 3-10 against *A. flavus* on peanut kernels was deserving of further study.

The objectives of this study were as follows: (i) to evaluate the efficacy of *S. yanglinensis* isolate 3-10 in inhibiting the growth of *A. flavus in vitro* and *in vivo* using peanuts as substrates; (ii) to determine the effect of *S. yanglinensis* on the biosynthesis of aflatoxin AFB_1_ and expression of *aflR* and *aflS* in *A. flavus*; and (iii) to investigate the mechanisms of the antifungal substance (AFS) of *S. yanglinensis* for suppression of *A. flavus* using electron microscopy.

## Materials and Methods

### Microorganisms and Media

Two microorganisms, *S. yanglinensis* 3-10 and *A. flavus* NRRL 3375, were used in this study. *S. yanglinensis* 3-10 was originally isolated from a healthy rice leaf grown in the field near Wuhan, China (Wan et al., 2008) and stored at -20°C. It was cultured on fermentation medium (soluble starch 3%, peptone 0.75%, yeast extract 0.025%, soybean meal 1%, K_2_HPO_4_⋅3H_2_O 0.5 g/l, KH_2_PO_4_ 0.7 g/l, MgSO_4_⋅7H_2_O 0.4 g/l, MnSO_4_⋅H_2_O 0.02 g/l, ZnSO_4_⋅7H_2_O 0.01 g/l) at 28°C for 72 h for AFS production (Shakeel et al., 2016). *A. flavus* NRRL 3375 was kindly provided by Dr. Desheng Qi of Huazhong Agricultural University in China and cultured on potato dextrose agar (PDA). PDA and PDB (potato dextrose broth) were prepared with peeled potato tubers using the procedures described by Fang (1998). Both microorganisms were incubated at 28°C.

### Preparation of the Culture Filtrates of *S. yanglinensis* 3-10 and Their Crude Extracts

The culture filtrate of *S. yanglinensis* 3-10 (CF^3-10^) was prepared by filtering a 3-day-old PDB shake culture through a 0.22-μm polycarbonate membrane filter, and the filtrate was extracted twice with ethyl acetate and dried in a vacuum to obtain total crude extract (CE^3-10^) (Shakeel et al., 2016). CE^3-10^ was dissolved in methanol at 12.5 mg/ml (w/v) and stored at 4°C for use as stock solution for subsequent tests.

### Effects of Metabolites of *S. yanglinensis* 3-10 on Mycelial Growth of *A. flavus* on PDA

Dual culture was used to evaluate the potential antagonism of *S. yanglinensis* 3-10 against *A. flavus.* These were placed in a 9-cm-diameter Petri dish containing 20 ml PDA. An aliquot of 0.5 ml of spore suspension of *S. yanglinensis* (1 × 10^8^ spores/ml) was streaked on one side of the plate, at least 3 cm from the center. After 24 h, a 6-mm-diameter agar plug from the leading edge of a 7-day-old culture of *A. flavus* was placed on the other side of the plate, 3 cm from the center. Plates without *S. yanglinensis* were used as control. The inoculated plates were incubated at 28°C. After 10 days, the colony diameter in each dish was measured.

PDA in Petri dishes amended with CF^3-10^ or CE^3-10^ was used to assess the effects of CF^3-10^ and CE^3-10^ on mycelia growth of *A. flavus.* In the CF^3-10^ treatment, the culture filtrate was amended into PDA to the final concentrations of 0, 0.5, 1.0, 1.5, 2.0, 2.5, or 3.0% (v/v), while fresh PDB was added to PDA as controls. In the CE^3-10^ treatment, the culture extract was added to PDA at final concentrations of 0, 0.5, 1.0, 1.5, 2.0, 2.5, or 3.0 μg/ml, while methanol was added to PDA at 0.2% (v/v) as the control. Mycelial plugs (6 mm diameter) of *A. flavus* cut from 7-day-old colonies were placed in the center of each dish. Plates were incubated at 28°C, with five replicate plates per treatment. Fungal growth was recorded after 7 days. Inhibition of growth (IG) of *A. flavus* by AFS of *S. yanglinensis* was calculated using the formula: IG (%) = (D_CK_ - D_3-10_)/D_CK_ × 100%, where D_CK_ represents the colony diameter in the treatment of control, D_3-10_ represents the colony diameter after treatment with CF^3-10^ or CE^3-10^. The experiments were repeated three times.

### Suppression of AFB_1_ Production in PDB by *A. flavus* Using *S. yanglinensis* 3-10 and Its AFS-Containing Products

To evaluate the effect of *S. yanglinensis* 3-10 on suppression of AFB1 production in PDB by *A. flavus*, spore suspensions of *S. yanglinensis* (S^3-10^) and AFS-containing products (CF^3-10^ and CE^3-10^) were tested. In the S^3-10^ treatment, *S. yanglinensis* was grown with *A. flavus* in 250 ml-Erlenmeyer flasks containing 50 ml PDB. The 50 ml PDB medium in 250 ml flasks was inoculated with 2.5 ml of spore suspension of *A. flavus* containing 1 × 10^8^ spores/ml, and 2.5 ml of spore suspension of *S. yanglinensis* (S^3-10^) at 1 × 10^8^ spores/ml. In AFS treatments, CF^3-10^ and CE^3-10^ were aseptically dispensed, before being inoculated with 2.5 ml of a 1 × 10^8^ spores/ml spore suspension of *A. flavus*. The final concentrations of metabolites of *S. yanglinensis* were 1.25, 2.5, 3.75, 5% (v/v) for the CF^3-10^ treatment, and 1.25, 2.5, 3.75, 5 μg/ml for the CE^3-10^ treatment. PDB inoculated with 2.5 ml of spore suspension of *A. flavus* (1 × 10^8^ spores/ml) was used as the control. All treatments (S^3-10^, CF^3-10^, CE^3-10^, and control) cultures were incubated at 28°C on a 150 rpm rotary shaker for 7 days, and then analyzed for fungal growth inhibition by weighted mycelial biomass of *A. flavus*, and AFB_1_ production with an aflatoxin plate kit (Agra Quant^®^ Aflatoxin B1 assay, COKAQ8000/COKAQ8048, Romer Labs Singapore Pte Ltd.). There were five flasks (replications) for each treatment, and the experiment was repeated three times.

### Antifungal Activity on Peanuts Under Storage Conditions

The inhibitory effect on *A. flavus* was determined following Zhang et al. (2013), with slight modifications. Peanut kernels with skins (cultivar Zhonghua No. 16) were surface sterilized with 5% NaOCl for 1 min and rinsed three times in sterilized water. In each Petri dish, 15 g peanut kernels were mixed with 2 ml of each treatment separately. The treatments were S^3-10^ (1 × 10^8^ spores/ml giving final 1.33 × 10^7^ spores/g peanut), CF^3-10^ (100%, v/v, giving final 0.133 ml/g peanut), and CE^3-10^ (100 μg/ml giving final 13.3 μg/g peanut). For the control treatment, 2 ml of sterile distilled water was added to 15 g of peanuts. There were five replicates dishes for each treatment. The plates were gently agitated by hand until the applied solutions were visibly absorbed by the kernel skins. Next, the kernels were inoculated with 1 ml of *A. flavus* spore suspension (1 × 10^8^ spores/ml), and again agitated until absorption of all liquid. After incubation at 28°C for 7 days, the growth of *A. flavus* on kernels was evaluated visually. The amount of AFB_1_ associated with peanut kernels in each treatment was determined following the method stated above. The experiments were repeated three times.

### Effect of *S. yanglinensis* 3-10 and Its AFS-Containing Products on *aflR* and *aflS* Expression

The effect of *S. yanglinensis* 3-10 metabolites on expression of AFB_1_ genes in *A. flavus* was studied with qRT-PCR. A spore suspension (2.5 ml) of *A. flavus* (1 × 10^8^ spores/ml) was added to 50 ml PDB and incubated for 7 days with either S^3-10^ (5 × 10^6^ spores/ml), CF^3-10^ (5%, v/v), or CE^3-10^ (5 μg/ml). As control *A. flavus* (2.5 ml, 1 × 10^8^ spores/ml) was grown without any filtrates or extracts from *S. yanglinensis*. Mycelia were collected by centrifugation and total RNA was extracted using a Trizol method described by Lou et al. (2015). For quantitative and qualitative analysis of total RNA, the A260/A280 ratio was determined, and gel electrophoresis was performed. cDNA was synthesized by using cDNA Synthesis SuperMix of TransGen Biotech according to instructions of the manufacturer. qRT-PCR was performed by using UltraSYBR Mixture of CWBIO where the 18S sequence was used as internal control. Previously designed primers were used for amplification of 18S rRNA, *aflR* and *aflS* in qRT-PCR (Kong et al., 2010). Fungal mycelium grown in the absence of *S. yanglinensis* spores and products was used as a control, and relative quantification was accomplished by using the delta Ct method described by Lou et al. (2015). The experiment was repeated three times.

### Scanning Electron Microscopy

Agar plugs of *A. flavus* were placed onto sterile cellophane films overlying PDA or PDA amended with CF^3-10^ 2% final concentration in Petri dishes, with one plug per dish, and three replicate dishes per strain. The dishes were incubated at 28°C for 7 days. Then, small cellophane film pieces (2 mm × 2 mm) colonized by mycelia of *A. flavus* were sampled from the center of each colony and fixed at 4°C for 12 h in 2% (w/v) glutaraldehyde in 0.1 M phosphate buffer (PB; pH 7.0). The mycelial specimens were dehydrated in graded ethanol. After drying in a critical point dryer (Model 13200-AB, SPI SUPPLIES, PA, United States) and gold-coating in a sputter coater (Model JFC-1600, NTC, Japan), the mycelial specimens were examined under a scanning electron microscope (Model JSM-6390/LV, NTC, Japan).

### Transmission Electron Microscopy

Morphology of the *A. flavus* samples was studied using transmission electron microscopy (TEM). Agar plugs, 6 mm in diameter, from 7-day-old culture were inoculated in the center of a piece of sterilized cellophane film (8 cm diameter) placed on PDA or PDA amended with CF^3-10^ at 2% in Petri dishes, with one plug per dish and three dishes per treatment. The dishes were incubated at 28°C for 7 days. The colonized films were cut into small pieces (3 mm × 3 mm) using a sharp razor. The cellophane film pieces were fixed in 2% (w/v) glutaraldehyde in 0.1 M PB (pH 7.0) at 4°C overnight. They were then washed in PB three times at room temperature (20–25°C), 10 min each time, and postfixed for 2 h in 1% osmium tetroxide, and stained for 1 h in 5% uranyl acetate (w/v) in 50% (v/v) of ethanol. Then, the fixed mycelial specimens were dehydrated in a graded series of ethanol, infiltrated with SPI-812 embedding medium and polymerized at 60°C for 12 h. Thin sections (50–60 nm) were cut with an ultra-microtome, mounted on copper grids, stained with 2% uranyl acetate and 5% aqueous lead citrate, and examined with a Hitachi transmission electron microscope (H-7650; Hitachi, Tokyo, Japan) at 80 kv. Images were recorded with a 4 KCCD camera (Model 832 ORIUS, Gatan, Pleasanton, CA, United States). At least 10 ultra-thin sections from each treatment were observed under TEM.

### Data Analysis

All data were analyzed by one-way analysis of variance (ANOVA) with the statistical software SAS v. 9.1 (SAS Institute Inc., Cary, NC, United States). To meet the requirements of homogeneity of variance, the percent inhibition growth data of *A. flavus* by AFS of *S. yanglinensis* 3-10 was arcsin-transformed to angular data prior to ANOVA. Differences were analyzed by the least significant difference (LSD) *post hoc* test at α = 0.05. After each analysis, mean values were individually back-transformed to numerical values.

## Results

### Effects of *S. yanglinensis* 3-10 on Mycelial Growth of *A. flavus in Vitro*

**FIGURE 1:**
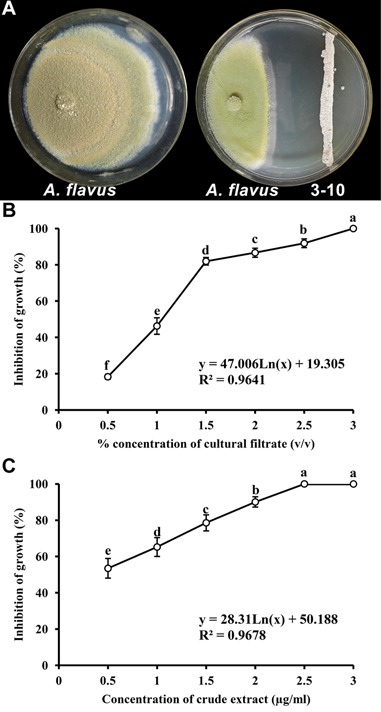
Inhibition of mycelial growth of *Aspergillus flavus* by *Streptomyces yanglinensis* 3-10. **(A)** Inhibitory effect of *S. yanglinensis* 3-10 against *A. flavus* in dual culture on PDA (28°C, 10 days). **(B)** Effect of culture filtrate of *S. yanglinensis* 3-10 on inhibition of growth of *A. flavus.* **(C)** Effect of crude extract of *S. yanglinensis* 3-10 on inhibition of growth of *A. flavus*. Means (circles) ± SD (whiskers) labeled with the same letter are not significantly different (*P* > 0.05) according to the least significant difference test.

The *S. yanglinensis* isolate 3-10 displayed significant inhibitory effects on the mycelial growth of *A. flavus* in dual culture *in vitro* assays. The average growth inhibition zone was 38.2 mm (**Figure 1A**). Compared to the control, the mycelial growth of *A. flavus* was reduced by 41%. In the tests on PDA plates, different concentrations of CF^3-10^ or CE^3-10^ incorporated in the PDA exhibited differential inhibitory effects on *A. flavus*. The efficacy (*Y_CF_*) of inhibition of mycelial growth of *A. flavus* by the cultural filtrates of *S. yanglinensis* was positively related to the concentration of the cultural filtrates (*X_CF_*) incorporated in PDA. *Y_CF_* = 47.006 Ln (*X_CF_*) + 19.305 (*r* = 0.9819, *P* < 0.01). With the increase of the concentration of the *S. yanglinensis* filtrate from 0.5 to 1.5%, the percentage inhibition of mycelial growth of *A. flavus* increased rapidly from 18.3 to 82.0% (**Figure 1B**). When the concentration of the filtrate was increased to 2 and 2.5%, the percentage inhibition slowly increased to 86.7 and 91.9%, respectively (**Figure 1B**). When the concentration of the filtrate was increased to 3%, the growth of *A. flavus* was completely inhibited (**Figure 1B**). The inhibition of mycelial growth of *A. flavus* by CE^3-10^ had a similar trend, yielding the equation *Y_CE_* = 28.311 Ln (*X_CE_*) + 50.188 (*r* = 0.9838, *P* < 0.01) (**Figure 1C**). When the concentration of the CE^3-10^ was increased to 2.5%, the growth of *A. flavus* was completely inhibited (**Figure 1C**).

### Suppression of *A. flavus* Mycelial Growth and AFB1 Production by AFS of *S. yanglinensis* 3-10

The antifungal and anti-aflatoxigenic activities of *S. yanglinensis* and its AFS-containing products were analyzed using mycelial dry weights and AFB_1_ production by *A. flavus*. Inhibition of *A. flavus* mycelial growth and aflatoxin production in liquid culture were positively correlated with the concentration of different treatments. The average dry weight of *A. flavus* was 767.9 mg per flask in the negative control. With the increased concentration ranging from 1.25 to 5% for CF^3-10^, or from 1.25 to 5 μg/ml for CE^3-10^, the dry weight of *A. flavus* decreased significantly from 119.9 to 62.1 mg per flask (CF^3-10^) and 169 to 63.4 mg per flask (CE^3-10^), while in S^3-10^ it was 520 mg per flask (**Table 1**). The growth inhibition percentage of *A. flavus* in CF^3-10^ (5%, v/v) and CE^3-10^ (5 μg/ml) increased dramatically to 91.9 and 91.7%, respectively, but only 32.3% in S^3-10^ (**Table 1**). Similar trends were observed in suppression of AFB_1_. Reduction of AFB_1_ production ranged from 52.4 to 93.4% in CF^3-10^ treatment and from 48.5 to 79.4% in CE^3-10^ treatment, while it was only 46.41% in S^3-10^ (**Table 1**).

**Table 1:** Inhibition of mycelium and aflatoxin production from *A. flavus* by *Streptomyces yanglinensis* 3-10 and its metabolites in PDB.

Treatments	Mycelial dry weight (mg)	Inhibition of growth (%)	AFB_1_ (μg/l)	Inhibition of AFB_1_ (%)
*A. flavus* alone	767.9 ± 24.4 a^∗^	–	121.48 ± 2.80 a	–
*Streptomyces* 3-10 spores + *A. flavus*	520 ± 18.3 b	32.28 f	65.10 ± 5.40 b	46.41 f
Culture filtrate (1.25%) + *A. flavus*	119.9 ± 7.3 d	84.39 d	57.83 ± 2.18 c	52.40 e
Culture filtrate (2.5%) + *A. flavus*	94.6 ± 6.3 ef	87.68 bc	46.88 ± 2.93 d	61.41 d
Culture filtrate (3.75%) + *A. flavus*	77.6 ± 6.3 fg	89.89 ab	30.04 ± 3.45 f	75.27 b
Culture filtrate (5%) + *A. flavus*	62.1 ± 4.9 g	91.91 a	8.04 ± 2.89 g	93.38 a
Crude extract (1.25 μg/ml) + *A. flavus*	169 ± 20.6 c	77.99 e	62.62 ± 7.92 bc	48.45 ef
Crude extract (2.5 μg/ml) + *A. flavus*	109.9 ± 7.3 de	85.69 cd	51.82 ± 1.84 d	57.34 d
Crude extract (3.75 μg/ml) + *A. flavus*	76.9 ± 9.4 fg	89.99 ab	35.42 ± 2.68 e	70.84 c
Crude extract (5 μg/ml) + *A. flavus*	63.4 ± 3.5 g	91.74 a	25.06 ± 4.1 f	79.37 b

*^∗^Mean values within the same column for each treatment without a letter in common are significantly different (*P* ≤ 0.05) according to the least significance test.*

### Antifungal Activity on Peanuts Under Storage Conditions

**FIGURE 2:**
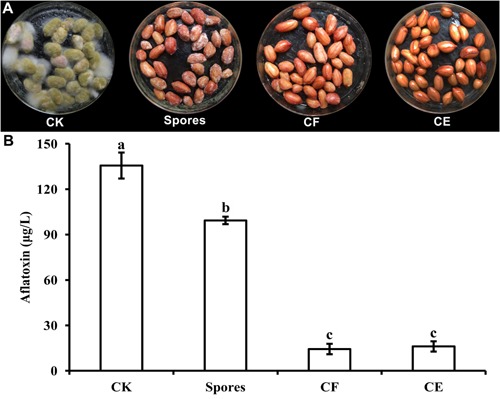
*Streptomyces yanglinensis* 3-10 and its AFS containing products inhibits *A. flavus* growth **(A)** and aflatoxin accumulation **(B)** in peanut kernels. CK, 1 × 10^8^ spores/ml of *A. flavus* alone; Spores, washed cell mixture 1 × 10^8^ spores/ml; CF, culture filtrate; CE, crude extract (100 μg/ml) (application volume 2 ml per 15 g peanut kernels). Treatment means (bars) ± SD (whiskers) labeled with the same letter are not significantly different (*P* > 0.05) according to the least significant difference test.

The growth of *A. flavus* on fresh peanuts was examined after incubation for 7 days in the presence of *S. yanglinensis* and its AFS-containing products (**Figure 2A**). Compared with the control treatment (inoculated with *A. flavus* alone), growth of *A. flavus* was completely inhibited in peanuts treated with CF^3-10^ (100%, v/v) or CE^3-10^ (100 μg/ml). There was no visible growth of *A. flavus* on peanut kernels, while the S^3-10^ treatment had visible white mycelium on the kernels. Furthermore, in the CF^3-10^ and CE^3-10^ treatments, the amount of detected AFB_1_ was 14.4 and 16.1 μg/l, respectively, much lower than in the control treatment (135.6 μg/l). This result shows that both AFS products, CF^3-10^ and CE^3-10^, were very effective in suppressing not only growth but also AFB_1_ production by *A. flavus*. Surprisingly, S^3-10^ was not as effective as the other two treatments. The amount of detected AFB_1_ in the S^3-10^ treatment was 99.4 μg/l, which was still significantly lower than the in untreated control treatment (**Figure 2**).

### Effect of *S. yanglinensis* 3-10 and Its AFS Containing Products on *aflR* and *aflS* Genes Expression

**FIGURE 3:**
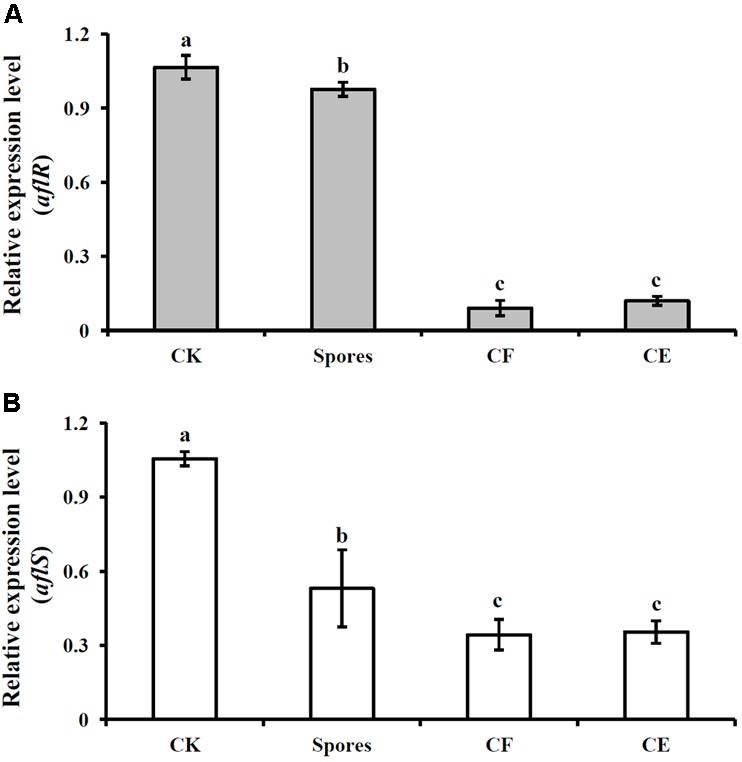
Expression of two aflatoxin biosynthesis genes *aflR* **(A)** and *aflS* **(B)** after treatment with *S. yanglinensis* 3-10 or its AFS containing products. The results are representative of the three independent experiments with similar results. CK, 5 × 10^6^ spores/ml of *A. flavus* alone; Spores, washed cell 5 × 10^6^ spores/ml; CF, culture filtrate (5%, v/v); CE, crude extract (5 μg/ml). Treatment means (bars) ± SD (whiskers) labeled with the same letter are not significantly different (*P* > 0.05) according to the least significant difference test.

In the control treatment (inoculated with *A. flavus* alone), the relative mRNA levels of *aflR* and *aflS* genes were 1.07 ± 0.12 and 1.06 ± 0.06, respectively. However, in the treatments of S^3-10^, CF^3-10^, and CE^3-10^, the expression levels of *aflR* were 0.98 ± 0.03, 0.09 ± 0.03, and 0.12 ± 0.02, respectively (**Figure 3A**). Similar trends were observed for the expression of *aflS*, at 0.58 ± 0.06, 0.34 ± 0.06, and 0.35 ± 0.05, respectively (**Figure 3B**). These results showed that *S. yanglinensis* could significantly suppress the expressions of these two genes (*P* < 0.05).

### Scanning Electron Microscopy

**FIGURE 4:**
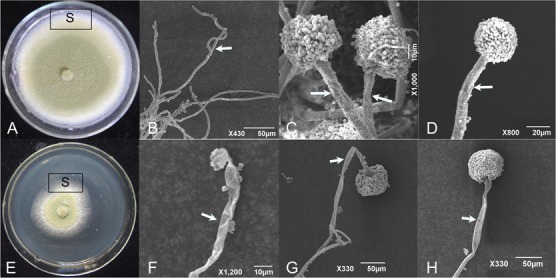
Scanning electron micrographs showing the healthy **(A–D)** and shriveled conidiophores **(E–H)** of *A. flavus* on PDA amended with culture filtrates of *S. yanglinensis* 3-10 at 2% (v/v). S, sampling site. The arrows mean hyphae or conidiophore.

*Aspergillus flavus* was cultured on PDA amended with CF^3-10^ at 2% (v/v) and the effects on mycelial and conidiophore morphology of *A. flavus* was observed under SEM. In untreated controls (unamended PDA), development of mycelium and conidiophore was normal with abundant conidia (**Figures 4A–D**). While in CF^3-10^ (2%, v/v) treated culture, the development of *A. flavus* was suppressed. Under SEM, conidiophore development was obviously abnormal, where mycelia and conidiophores were shriveled compared to untreated controls (**Figures 4E–H**).

### Transmission Electron Microscopy

**FIGURE 5:**
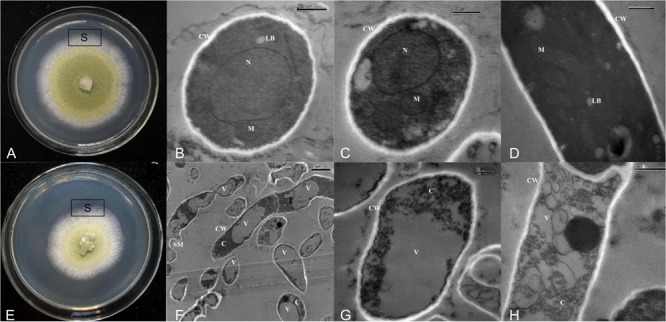
Transmission electron micrographs showing normal development of cell organelles **(A–D)** and disintegration of cytoplasm and cell organelles **(E–H)** when *A. flavus* was cultured on PDA amended with culture filtrates of *S. yanglinensis* 3-10 at 2% (v/v). VV, SM, N, M, LB, CW, C, vacuole; SM, shriveled mycelium; N, nucleus; M, mitochondria; LB, lipid bodies; CW, cell wall; C, cytoplasm; S, sampling site.

Thin sections examined by TEM showed that in control cultures, *A. flavus* possessed all the components of healthy eukaryotic cells (**Figures 5A–D**). But in cultures treated with CF^3-10^ (2%, v/v), only large vacuoles and disintegration of cytoplasm were observed (**Figures 5E–H**). In control treatments, there were fungal cells with maximum electron density and normal development of cell organelles (**Figures 5B–D**), while in cultures treated with CF^3-10^ (2%, v/v), the hyphae were degenerated and collapsed. The cell walls were well preserved and frequently visible (**Figures 5F–H**). Healthy cells contained obvious mitochondria and lipid bodies, while in treated cultures, development of these organelles was inhibited. In treated cultures, the lipid bodies were large and abundant and appeared to compress the cytoplasm content in the vicinity of plasma membranes making it difficult to recognize other cell organelles (**Figures 5F–H**). In a few healthy cells, single large nuclei were visible (**Figures 5B,C**), but because of the development of large vacuoles in treated cultures, the presence of nuclei was not obvious (**Figures 5F–H**).

## Discussion

*Streptomyces yanglinensis* isolate 3-10 was effective in suppressing postharvest disease caused by *A. flavus* both *in vitro* (liquid or solid media) and *in vivo* on peanut kernels. The cells of *S. yanglinensis* had inhibitory effects on *A. flavus* and the metabolites of *S. yanglinensis* were even more strongly inhibitory to *A. flavus* on PDA and in PDB. A comparison of our results with previous studies on *A. flavus* inhibition revealed that our compounds showed greater inhibitory effects than Bacillomycin D (Zhang et al., 2008) or extracts of *Agave asperrima* and *Agave striata* (Eduardo et al., 2005).

Our results are in agreement with those reported from other antagonists such as *Streptomyces* sp. ASBV-1 (Zucchi et al., 2008), *Streptomyces hygroscopicus* (Zhang et al., 2013), *Streptomyces* VITSVKS spp. (Kumar and Kannabiran, 2010), and *Streptomyces* VITSTK7 (Thenmozhi et al., 2013), which suggest the accumulation of bioactive metabolites in cultural medium or AFS production. Many *Streptomyces* isolates show antifungal activity against *A. flavus*, and can reduce aflatoxin B1 residual concentration, but the effectiveness depended on the particular isolate (Verheecke et al., 2014). Sultan and Magan (2011) reported that crude extracts of *Streptomyces* strain AS1 could achieve more than 85% inhibition of mycelial growth of *A. flavus* at 50 μg/ml, but in our results, the mycelial growth of *A. flavus* was completely inhibited by crude extracts of *S. yanglinensis* at 2.5 μg/ml. Results from the *in vivo* tests showed that the CF^3-10^ was as effective as the CE^3-10^, and even more effective in some cases. The concentration of the antagonist had significant effects on biocontrol effectiveness, and the higher the concentration of *S. yanglinensis* or its products were, the higher was the activity. The highest biocontrol activity was evident with CF^3-10^ rather than CE^3-10^ (100 μg/ml), indicating that some unknown bioactive metabolites could not be extracted with ethyl acetate.

Our findings also showed that *S. yanglinensis* was able to significantly inhibit the biosynthesis of aflatoxins in PDB and on peanut kernels (*P* < 0.05). We found that the biosynthesis of aflatoxins was related to the expression of aflatoxin pathway regulatory genes including *aflR* and *aflS*, as has been previously reported (Flaherty and Payne, 1997; Meyers et al., 1998). Quantitative PCR showed that in the *aflS* knockout mutants, the lack of *aflS* transcript led to a 5- to 20-fold reduction of expression of some aflatoxin pathway genes such as *aflC* (*pksA*), *aflD* (*nor-1*), *aflM* (*ver-1*), or *aflP* (*omtA*). The mutants lost the ability to synthesize aflatoxin intermediates and no aflatoxins were produced (Meyers et al., 1998). Deletion of *aflR* in *Aspergillus parasiticus* abolished the expression of other aflatoxin pathway genes (Cary et al., 2000). Overexpression of *aflR* in *A. flavus* up-regulated aflatoxin pathway gene transcription and aflatoxin accumulation (Flaherty and Payne, 1997), which was in accordance with a previous report in *A. parasiticus* (Chang et al., 1995). Our findings are in agreement with previous studies which demonstrated that *aflS* might be involved in the regulation of aflatoxin biosynthesis through the regulation of other genes, while *aflR* was more directly involved (Amare and Keller, 2014). However, to uncover the complete antagonistic physiological activities of *S. yanglinensis* and the underlying mechanisms, a more thorough investigation of all aflatoxin pathway genes should be investigated by the use of whole genome gene expression analyses such as RNA-Seq and differential gene expression, followed by specific quantification with targeted gene primers in real-time qPCR.

Lyu et al. (2017) identified active antifungal compounds purified from the crude extract of *S. yanglinensis* as reveromycins A and B. Reveromycins A is the main active AFS in the crude extract, which accounts for 37.7% of the crude extract. And the antifungal activity of reveromycins A is higher than that of reveromycins B (Lyu et al., 2017). Reveromycins A has been found to have a variety of effects, including inhibitory effects on mitogenic activity induced by epidermal growth factors (Osada et al., 1991), production of hormone-dependent tumors (Takahashi et al., 1997), induced proliferation of *Candida* species (Takahashi et al., 1992; Fremlin et al., 2011; Osada, 2016), and inhibition of mycelial growth of plant pathogenic fungi like *Botrytis cinerea, Mucor hiemails, Rhizopus stolonifer*, and *Sclerotinia sclerotiorum* (Lyu et al., 2017). Further research is needed on environmental effects and safety of reveromycins A, and on its antifungal activity against *A. flavus* and other food storage fungi.

## Conclusion

In conclusion, this study demonstrated that *S. yanglinensis* isolate 3-10 has the potential for controlling peanut kernel postharvest disease caused by *A. flavus* and reducing the accumulation of aflatoxin. Still necessary are further studies to evaluate the potential risks of *S. yanglinensis* and its AFS for controlling *A. flavus* in peanut kernels and other agricultural commodities, as well as practical methods for delivery of optimal forms and concentrations of the antagonistic substances.

## Author Contributions

QS, GL, and LY designed the research; QS and AL performed the research; QS, MW, JZ, and LY analyzed the data; QS, TH, and LY wrote the paper.

## Conflict of Interest Statement

The authors declare that the research was conducted in the absence of any commercial or financial relationships that could be construed as a potential conflict of interest.
